# Septic Shock, Infective Endocarditis, Septic Embolization and Disseminated Intravascular Coagulation Caused by a Toxigenic *C. diphtheriae* Strain: A Case Report

**DOI:** 10.3390/healthcare14131890

**Published:** 2026-06-29

**Authors:** Matteo Fabris, Ivan Martinello, Flavio Bassi

**Affiliations:** Department of Emergency, “Santa Maria della Misericordia” University Hospital of Udine, Azienda Sanitaria Universitaria Friuli Centrale, 33100 Udine, Italy

**Keywords:** *Corynebacterium diphtheriae*, disseminated intravascular coagulation, infective endocarditis, septic shock, toxigenic strain

## Abstract

**Background:** Diphtheria is an acute infectious disease caused by *Corynebacterium diphtheriae*. Despite several worldwide outbreaks, it is now considered a rare disease by industrialized countries. Clinical manifestations usually account for oropharyngeal lesions, but rare cases of systemic involvement (mainly endocarditis) have been described among non-toxigenic strains. **Case description:** We report the case of a patient who experienced septic shock, disseminated intravascular coagulation and multiorgan failure due to *Corynebacterium diphtheriae* infection. The pathogen was further characterized as a highly toxigenic strain. Infective endocarditis with mitral and aortic valve vegetations led to early multiorgan septic embolization. Major stroke, liver function impairment, heart failure and acute kidney injury were the main findings. Unlike the typical forms of infection caused by this pathogen, there was no evidence of airway or skin involvement. Furthermore, apart from hemocultures, none of the other investigations (pharyngeal swabs, bronchoalveolar lavages, urine culture) ever tested positive for the bacteria. **Conclusions:** The report we present describes a case of *C. diphtheriae* infection with many atypical characteristics: (i) lack of any pathognomonic signs or symptoms; (ii) extensive endocarditic process (very uncommon for toxigenic strains); (iii) early septic emboli development, with rapid evolution to multiorgan failure; (iv) detection of disseminated intravascular coagulation. Despite disseminated intravascular coagulation being a known complication of septic shock, regardless of the etiological agent, according to our literature research, this is the second known case driven by *C. diphtheriae* infection in an adult.

## 1. Introduction

Diphtheria is an acute infectious disease caused by *Corynebacterium diphtheriae*, a non-capsulated, Gram-positive bacillus [1].

The pathogen is commonly transmitted by droplets, although rare cases of contact transmission have been described [2]. Commonly, it causes laryngopharyngitis, distinguishable by the presence of pathognomonic pseudomembranes, which are greyish plaques with potential necrotic-hemorrhagic evolution, observable mainly in the palatine tonsils but potentially affecting the soft palate, hypopharynx and larynx as well [3]. Cutaneous diphtheria is otherwise characterized by indolent skin ulcers covered with pseudomembranes, and it tends to involve the limbs [2].

Exotoxin production is the primary cause of virulence. Not all strains are toxigenic; toxin production depends on the expression of a specific gene carried by a circular DNA molecule, acquired after a β-lysogenic phage interaction [2]. Exotoxin is also responsible for the potential systemic manifestations: myocarditis, cranial nerve peripheral paralysis and acute kidney injury (AKI) [3,4]. Despite that, toxigenic strains commonly cause respiratory symptoms, whereas cardiac function impairment, such as myocarditis and infective endocarditis (IE), is more likely to be attributable to non-toxigenic strains [1,4,5].

Although the global vaccination coverage rate has reached 86%, new outbreaks could still be observed. Considering that humans are the only reservoir of the pathogen, the status of healthy carriers might represent an important condition for the persistence of the disease [6].

Hereafter, we describe the case of a patient who developed early and extensive cardiac valve injuries, septic shock, multiorgan failure (MOF) and disseminated intravascular coagulation (DIC) after a toxigenic *C. diphtheriae* strain infection.

## 2. Case Presentation

Signed consent was acquired from the patient prior to the publication of this case report.

A 49-year-old male accessed the emergency department, complaining about persistent fever, rigor nucalis, headache and disabling arthromyalgias predominantly affecting wrists, hands, ankles and feet. He reported a recent trip to a foreign country but excluded any raw food consumption, contact with febrile people, or tick bites. Medical history only referred to mild psoriasis without ongoing therapy.

Psoriatic patches were seen on the elbows, without any other dermatological issues. Neither the cardiopulmonary physical examination nor the airway evaluation reported any lesion. Blood tests revealed hypokalemia (3.1 mM), neutrophilic leukocytosis (13,750 U/μL) and thrombocytopenia (70,000 U/μL) (Figure S1). A highly toxigenic *C. diphtheriae* strain was detected in hemocultures. Notably, all nasopharyngeal swabs performed tested negative. Furthermore, an otolaryngologist’s evaluation excluded pharyngeal pseudomembranes, as well as any other pharyngeal lesions. Similarly, the multiple optical bronchoscopies performed during hospitalization never showed any tracheal or bronchial injury.

The patient suddenly experienced left hemiplegia and aphasia. Computed tomography (CT) showed acute occlusion of the proximal tract of the right middle cerebral artery (MCA) (Figure 1). Given thrombocytopenia, intravenous fibrinolytic treatment was unfeasible due to an unacceptable hemorrhagic risk. Thus, thromboaspiration was carried out. Cultures from the sample material later tested positive for *C. diphtheriae*, assessing the diagnosis of septic cerebral embolism. Even though the interventional procedure was successful, further CT scans revealed an ischemic progression in MCA territories. Midline shift associated with compression of the ventricular system eventually led to decompressive craniectomy being performed (Figure S2).

CT scan was further extended to the abdomen to detect any other sites of embolization; two large ischemic areas were observed in the middle-upper third of the spleen and in the upper pole of the left kidney (Figure 2).

The persistent increase in troponin subunit levels (up to 1178 ng/mL) led to suspicion of cardiac involvement. Transesophageal echocardiography (TEE) was therefore performed. Severe insufficiency of the aortic valve was observed due to multiple vegetations and rupture of the right coronary cusp (Video S1). Two vegetations affecting both the atrial and ventricular sides of the mitral valve were detected as well. The major (17 mm) presented with central vacuolization and multiple perforations, which was the cause of severe valve insufficiency (Video S2).

Given MOF, treatment required a multidisciplinary approach. Once the pathogen was isolated from hemocultures and an antibiogram was obtained (Table 1), antibiotic therapy was started with piperacillin/tazobactam, clindamycin and gentamicin. After confirming brain septic emboli, meropenem was added to optimize central nervous system penetration. Given the characterization as a highly toxigenic strain, an intravenous diphtheria antitoxin infusion was also introduced.

Renal replacement therapy was started as well. Not only did it allow AKI therapy, but it also granted septic cytokine adsorption.

The mitral and aortic valves’ severe insufficiency required cardiac surgery for valve replacement. Intra-operative evaluation showed florid vegetations on all aortic valve cusps and a pseudo-aneurysmal cavity at the sub-commissural trigone between the right and left coronary cusps. Large vegetations with severe remodeling of the valve leaflets and sub-valvular apparatus were also observed on the mitral valve (Figure 3).

The patient was discharged to the ward on the 106th day of hospitalization (Figure S3).

## 3. Discussion

Diphtheria incidence has progressively decreased worldwide. This is attributable to increasing vaccination coverage, particularly following the introduction of the trivalent diphtheria–tetanus–pertussis vaccine (DTP) [7]. A fully completed DTP vaccination cycle (three doses plus one booster every ten years) can prevent 87% of symptomatic cases but does not prevent the infection [6]. The status of a healthy carrier might thus represent an important condition for disease transmission, despite patients with asymptomatic diseases having been shown to transmit the pathogen at 24% of the rate of symptomatic cases [6].

The patient described in our report correctly followed the infancy vaccination cycle but never underwent adulthood booster administration. This may justify the systemic evolution of the infection that he experienced. Interestingly, none of his relatives, with whom he lived in close contact at home, ever showed any symptoms. The vaccination status of the family members was not available.

*C. diphtheriae* is commonly transmitted by droplets and normally causes symptoms affecting oropharynx or upper airways. The lack of any ascertained source of infection makes our report atypical. No pathognomonic lesion (pseudomembranes) nor upper airway or cutaneous injuries have ever been detected. Furthermore, the patient did not manifest any risk factors (traumatic injuries, tick bites, journeys into endemic areas, contact with known febrile people).

Atypical presentation, lack of pathognomonic lesions and sudden clinical worsening led to a challenging diagnosis. Meningoencephalitis was suspected during the first medical visit due to migraine and rigor nucalis. Lumbar puncture was not performed because of prompt symptom regression after steroid and non-steroidal anti-inflammatory drug administration, absence of further typical semiotic signs and relative contraindication due to thrombocytopenia.

Unexpected cerebral thrombosis associated with thrombocytopenia, endocarditis and AKI in a known psoriatic patient aroused suspicion for lupus overlapping syndrome [8]. Such a diagnosis was further excluded due to the absence of circulating autoantibodies.

Before *C. diphtheriae* isolation, the evidence of massive valve vegetations led to a prompt search for IE’s most addressed pathogens. *Streptococcus pyogenes* was excluded by negative nasopharyngeal swabs and an antistreptolysin test. Staphylococcal infection was ruled out on both epidemiological and clinical criteria due to the absence of risk factors (such as drug abuse, cardiac implanted electronic devices, or recent surgery) and the lack of impairment of the right-side heart valves [9]. HACEK group pathogens were excluded due to the absence of any risk factors: congenital heart disease, periodontitis, polymicrobial wound infections, otitis media, or pharyngeal abscesses [10].

Non-toxigenic strains have previously been reported as a rare cause of IE, mostly in patients with known risk factors (intravenous drug abuse, prosthetic heart valves, preexisting heart abnormalities) [11]. Acute IE cases driven by toxigenic strains are instead anecdotal [12].

According to Mishara et al., patients with cardiac valve abnormalities (including prosthetic) and ongoing diphtheric endocarditis should undergo surgery as soon as possible (ideally within 48 h); on the other hand, patients with normal valves might be treated with proper antibiotic therapy alone if urgent surgery is not needed [13].

In contrast to the literature, our patient experienced an extensive mitral and aortic valve impairment driven by a toxigenic *C. diphtheriae* strain despite the absence of any predisposing factor. Cardiac surgery was already planned during the second day of ICU hospitalization but was postponed until the 38th day due to an excessive hemorrhagic risk.

The *C. diphtheriae* cellular adhesion mechanism has been largely studied but not yet fully understood. The main bonding factors appear to be adhesive pili, which mediate interaction between the pathogen and the host’s fibrinogen or collagen [14]. Further genomic studies showed that the expression of a single gene (spa—pilus gene cluster) may provide an advantage in colonizing different tissues in the host [15]. This evidence may indicate a different cellular tropism, possibly explaining the rapid and extensive damage to cardiac valves, but not to oropharynges, which was observed in our case.

Moreover, our report showed the simultaneous presence of thrombotic foci associated with thrombocytopenia. This raised suspicion for DIC. The Japanese Association for Acute Medicine score, which has been proven to be a reliable indicator of DIC in septic patients, was diagnostic for DIC when applied to our case [16] (Table 2). Although *C. diphtheriae* infection might represent a very rare cause of DIC in pediatric patients, according to our literature research, it has previously been reported only once in an adult [4,17].

## 4. Conclusions

This report describes a case of *C. diphtheriae* infection with many atypical characteristics: (i) development in a patient without any risk behavior apart from an incomplete adulthood immunization; (ii) absolute lack of typical signs and symptoms; (iii) fast and extensive aggression of cardiac valves driven by a toxigenic strain; (iv) early septic embolization; (v) rapid evolution to septic shock and MOF; (vi) DIC development. DIC is a known complication of septic shock, regardless of the etiological agent; however, according to our literature review, only one report previously described a case of DIC caused by *C. diphtheriae* infection in an adult.

These characteristics might be addressed by the pathogen’s anomalous expression of virulence factors, which might have given a peculiar tropism to the endocardium rather than oropharynx mucosal cells.

## Figures and Tables

**Figure 1:**
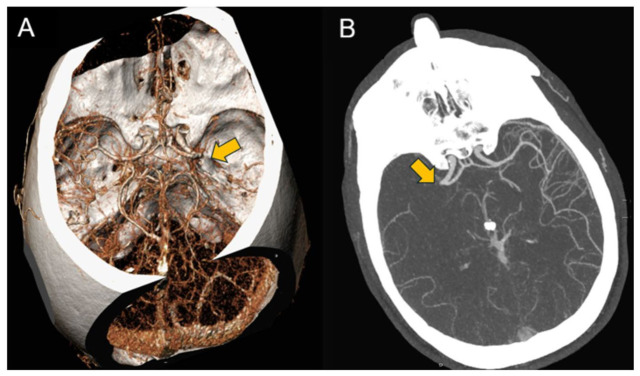
(**A**) Three-dimensional reconstruction of angiographic CT brain scan showing right MCA lack of perfusion (arrow) due to septic emboli. (**B**) CT brain scan, coronal axis, assessing the diagnosis of acute right MCA occlusion (note the lack of contrast-enhancement distal to the arrow).

**Figure 2:**
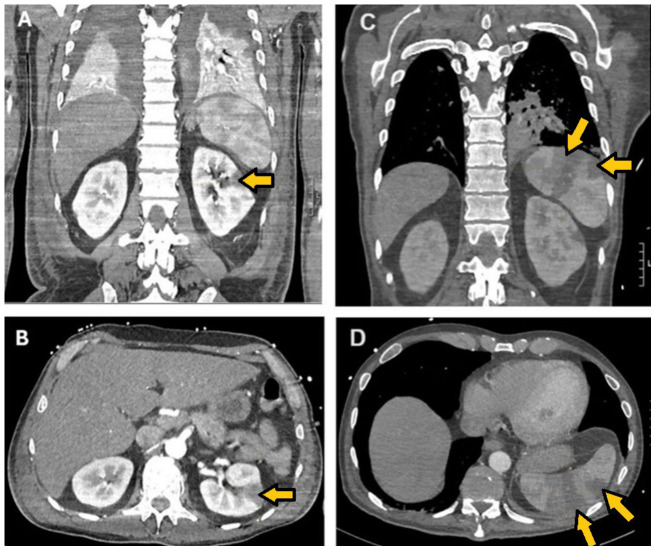
Multiple abdominal coronal (**A**,**C**) and transverse (**B**,**D**) scans assessing for the diagnosis of ischemic lesions due to septic emboli among left kidney (arrows at **A**,**B**) and upper left pole of spleen (arrows at **C**,**D**).

**Figure 3:**
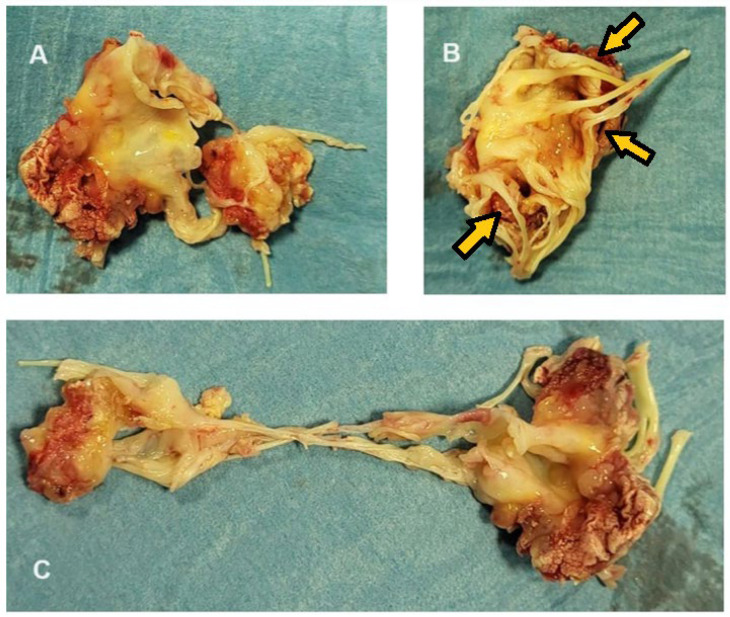
Intra-operative specimen of mitral valve: (**A**) atrial face showing a destructive process due to a massive vegetation; (**B**) ventricular side: intact chordae tendineae but extensive nodulations among peripheral surfaces of both cusps (arrows); (**C**) extensive vegetation affecting antero-septal cusp (left side of the image) and massive vegetations on atrial side of both cusps (right side of the image).

**Table 1:** Antibiogram obtained from hemocultures. S = sensitive; I = intermediate; R = resistant; MIC = minimal inhibitory concentration; CFU = colony-forming units.

Test: Hemoculture	Pathogen: *C. diphtheriae*	Concentration: 10^5^ CFU/mL
Antibiotic	MIC	Efficacy Profile
Amoxicillin	0.125	S
Cefotaxime	0.5	I
Ciprofloxacin	0.75	R
Clindamycin	0.38	S
Doxycycline	0.125	S
Erythromycin	0.047	S
Linezolid	0.19	S
Meropenem	0.064	S
G penicillin	0.094	I
Rifampicin	<0.002	S
Tetracycline	0.19	S
Trimethoprim/sulfamethoxazole	1.5	R

**Table 2:** Japanese Association for Acute Medicine score for disseminated intravascular coagulation diagnosis, and parameters seen in our report. INR = international normalized ratio; SIRS = systemic inflammatory response syndrome; WBC = white blood cells; bpm = beats per minute; MV = mechanical ventilation.

JAAM Score		Our Case Report	
Factors	Points	Parameters	Points
**Platelet count**			
<80 × 10^3^ u/μL or >50% decrease within 24 h	3	50 × 10^3^ u/μL	3
≥80 × 10^3^ and <120 × 10^3^ u/μL or 30–50% decrease between 24 h	1		
≥120.000	0		
**INR**			
≥1.2	1	1.32	1
<1.2	0		
**SIRS score:**			
- **WBC > 12 × 10^3^ u/μL or <4 × 10^3^ u/μL**	15 × 10^3^ u/μL		
- **Body temperature > 38 °C or <36 °C**	38.6 °C		
- **Heart rate > 90 bpm**	94 bpm		
- **Bradypnea or tachypnoea**	14 breaths/minute (MV)		
≥3 positive parameters	1	3 parameters	1
<3 positive parameters	0		
**Fibrin degradation products**			
D-dimer ≥ 25 μg/mL	3	2.75	0
D-Dimer 10–25 μg/mL	1		
D-dimer < 10 μg/mL	0		
**Total** (≥4 is diagnostic for DIC)			5

## Data Availability

The original contributions presented in this study are included in the article/Supplementary Materials. Further inquiries can be directed to the corresponding author.

## Supplementary Materials

The following supporting information can be downloaded at: https://www.mdpi.com/article/10.3390/healthcare14131890/s1, Figure S1: Graphical representation of infection/inflammation, renal, neurological and coagulation data trend during the first 70 days of hospitalization; Figure S2: Sequential brain CT scans assessing for ischemic brain lesions evolution; Figure S3: Timeline; Video S1: Transesophageal echocardiography, mid-esophageal section, short-axis; Video S2: Transesophageal echocardiography mid-esophageal section, long-axis.
