# Supplementary figures and images for: Septic Shock, Infective Endocarditis, Septic Embolization and Disseminated Intravascular Coagulation Caused by a Toxigenic C. diphtheriae Strain: A Case Report
Source: Healthcare (Basel). 2026 Jun 29;14(13):1890. doi: 10.3390/healthcare14131890 (PMC13362524; doi:10.3390/healthcare14131890)

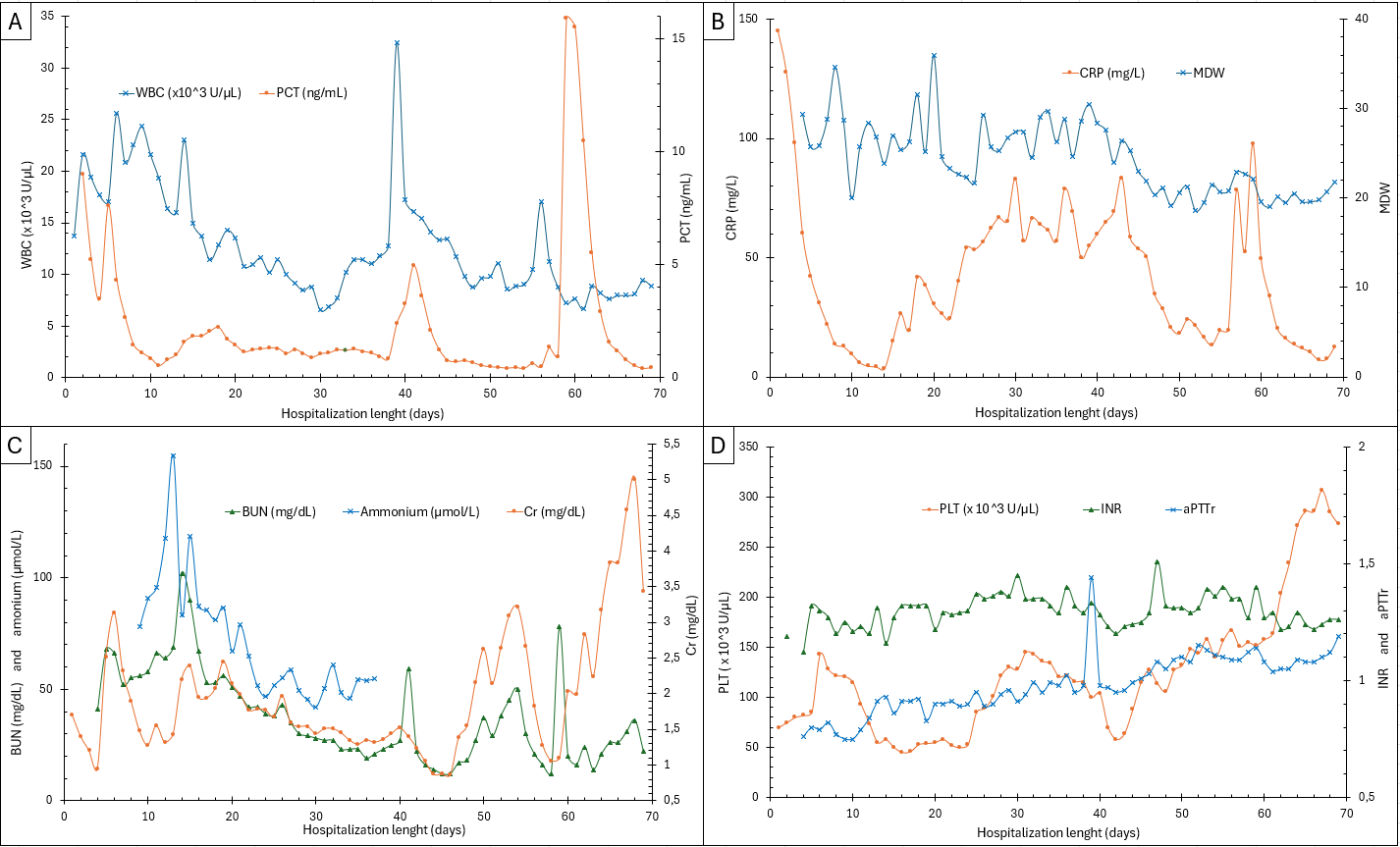

Supplement: Supplementary file 1 [file healthcare-14-01890-s001.zip › Figure S1.png]

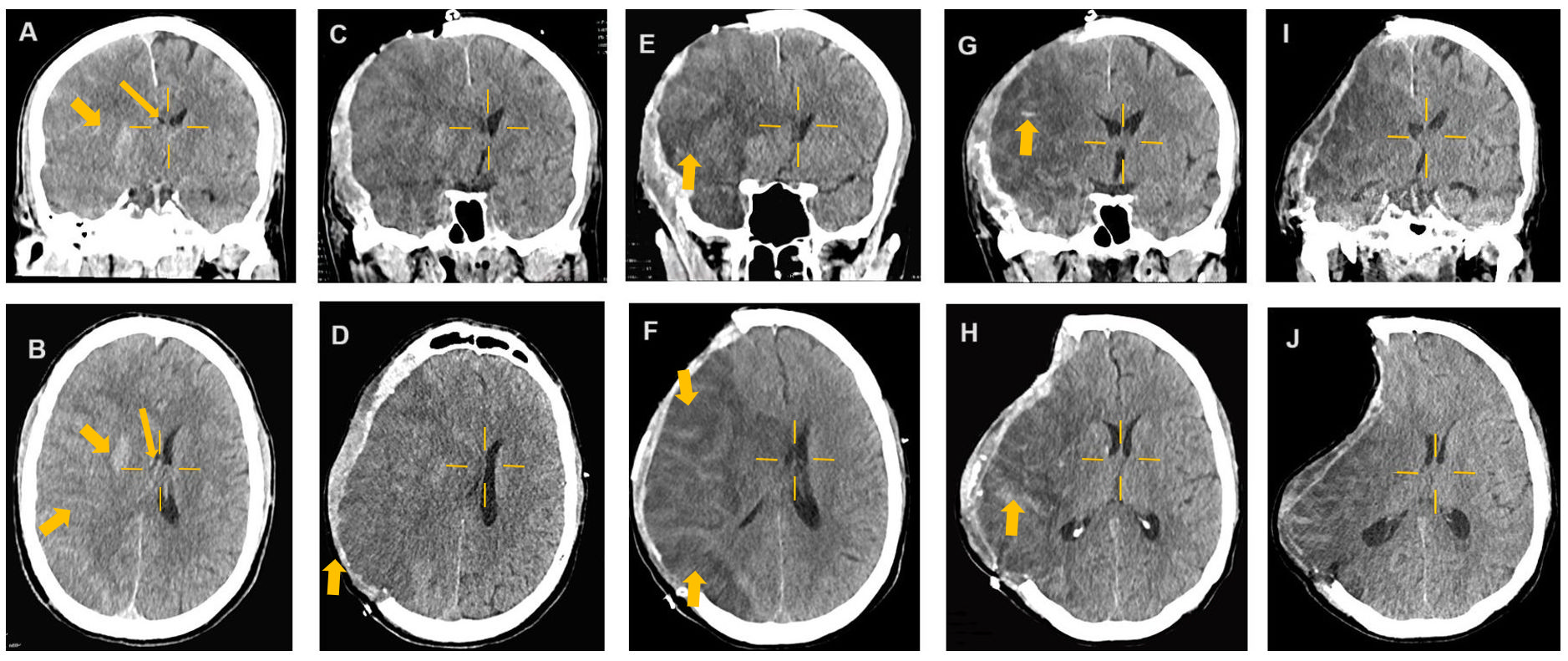

Supplement: Supplementary file 1 [file healthcare-14-01890-s001.zip › Figure S2.png]

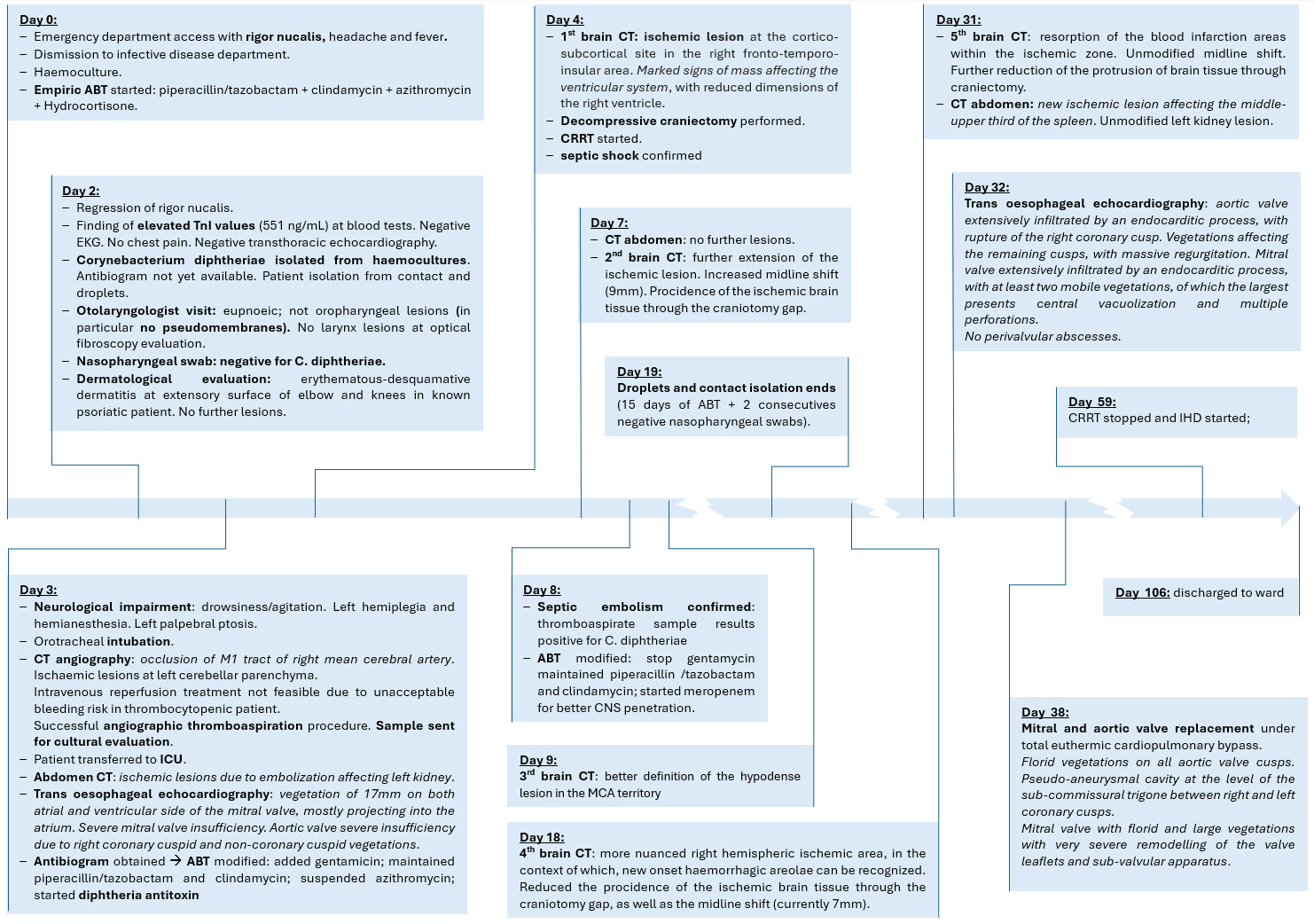

Supplement: Supplementary file 1 [file healthcare-14-01890-s001.zip › Figure S3.png]

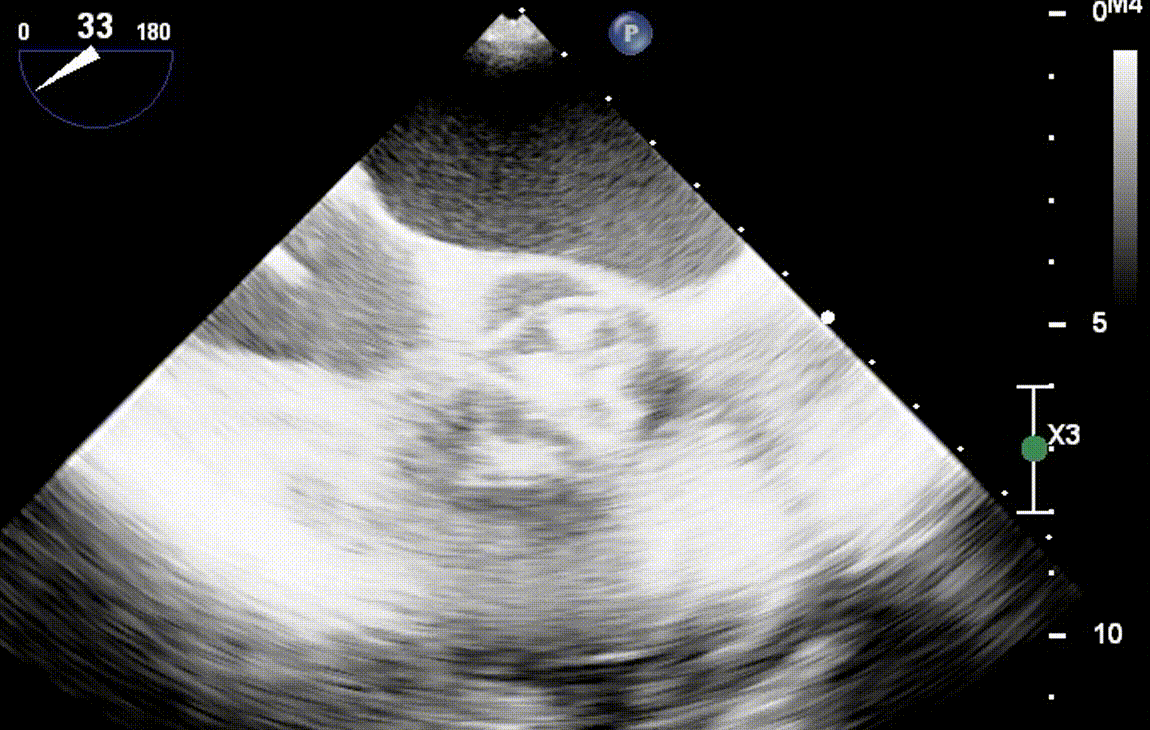

Supplement: Supplementary file 1 [file healthcare-14-01890-s001.zip › Video S1.gif]

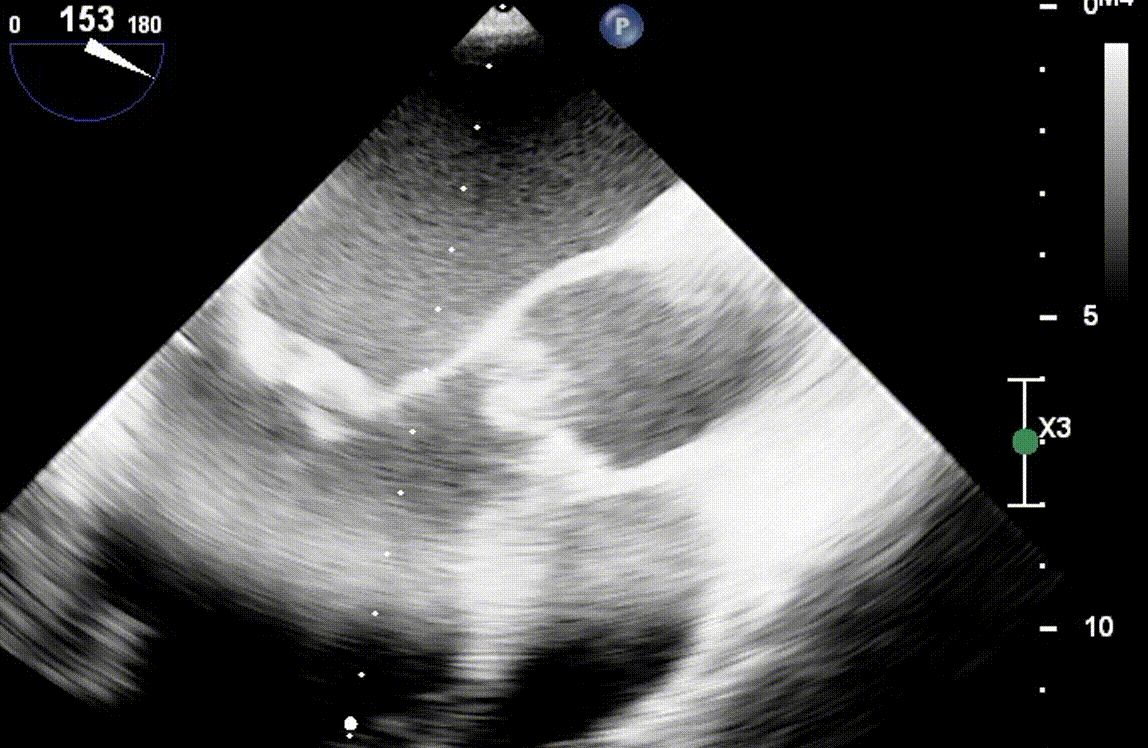

Supplement: Supplementary file 1 [file healthcare-14-01890-s001.zip › Video S2.gif]
